# Early postnatal home visits: a qualitative study of barriers and facilitators to achieving high coverage

**DOI:** 10.1186/s12889-018-5922-7

**Published:** 2018-08-29

**Authors:** Yared Amare, Pauline Scheelbeek, Joanna Schellenberg, Della Berhanu, Zelee Hill

**Affiliations:** 1 Consultancy for Social Development, PO Box 70196, Addis Ababa, Ethiopia; 2 London School of Hygiene and Tropical Medicine, Keppel Street, London, WC1E 7HT UK; 3 University College London, 30 Guilford Street, London, WC1N 1EH UK

**Keywords:** Newborn, Postnatal, Community health worker, Qualitative, Ethiopia

## Abstract

**Background:**

Timely interventions in the postnatal period are important for reducing newborn mortality, and early home visits to provide postnatal care are recommended. There has been limited success in achieving timely visits, and a better understanding of the realities of programmes is needed if improvements are to be made.

**Methods:**

We explored barriers and facilitators to timely postnatal visits through 20 qualitative interviews and 16 focus group discussions with families and Health Extension Workers in four Ethiopian sites.

**Results:**

All sites reported some inaccessible areas that did not receive visits, but, Health Extension Workers in the sites with more difficult terrain were reported to make more visits that those in the more accessible areas. This suggests that information and work issues can be more important than moderate physical issues. The sites where visits were common had functioning mechanisms for alerting workers to a birth; these were not related to postnatal visits but to families informing Health Extension Workers of labour so they could call an ambulance. In the other sites, families did not know they should alert workers about a delivery, and other alert mechanisms were not functioning well. Competing activities reducing Health Extension Worker availability for visits, but in some areas workers were more organized in their division of their work and this facilitated visits. The main difference between the areas where visits were reported as common or uncommon was the general activity level of the Health Extension Worker. In the sites where workers were active and connected to the community visits occurred more often.

**Conclusions:**

If timely postnatal home visits are to occur, CHWs need realistic catchment areas that reflect their workload. Inaccessible areas may need their own CHW. Good notification systems are essential, families will notify CHWs if they have a clear reasons to do so, and more work is needed on how to ensure notification systems function. Work ethic was a clear influencer on whether home visits occur, studies to date have focused on understanding the motivation of CHWs as a group, more studies on understanding motivation at an individual level are needed.

## Background

Approximately 2.9 million neonates die every year, which accounts for 44% of deaths among children under five years of age. 73% of these deaths are in the first week of life, and 36% on the first day [1, 2]. This highlights the importance of timely intervention in this vulnerable period [2]. Several life saving newborn behaviours can be promoted, and interventions delivered, through early postnatal care (PNC). These include an assessment of the baby and treatment or referral, and counselling on breastfeeding, thermal care, hygiene, cord care and on danger signs [3, 4].

Evidence shows that home visits by community health workers (CHWs) can be an effective means of delivering postnatal care in high mortality settings, and can reduce mortality [5, 6], and this strategy has been adopted by 59 of the 75 countries in the Countdown to 2015 report [7]. Observational data suggest that these visits need to occur within 2 days of delivery to be effective [8]. The World Health Organization recommends that those who deliver at home should receive a home visit within 24 h of delivery, and those who deliver in a facility should receive PNC in the facility for the first 24 h and home visits from day three [3].

There have been mixed results in achieving timely visits. Data from sub-Saharan Africa show modest coverage of postnatal care home visits by CHWs, even in study and pilot program settings. In Malawi only 11% of women received a PNC visit within 3 days of delivery, in Tanzania only 15% within 2 days, in Uganda 26% received a visit on day 1, and in Ghana 38% received a visit on day 1 or 2 (figures calculated from authors data) [6, 9–12]. Data from government programmes show even lower coverage levels [7]. Given the timing of newborn deaths and the importance of early visits, the need for research in this area has been acknowledged [13]. We identified only three quantitative studies exploring factors affecting the coverage of postnatal care home visits. A meta-analysis of quantitative data from Bangladesh, Malawi and Nepal found that early visits were more likely if a mother had been visited in pregnancy, if they had notified the CHW about the birth, and if the birth had been at home. In Ethiopia attending ANC, having more than two family meetings in pregnancy with a CHW, delivering with a CHW or skilled attendant, and having the CHW’s phone number were associated with receiving early home visits [14]. No association with maternal socio-demographic characteristics were found in any of the studies [10, 11, 14]. A program review that conducted qualitative interviews with government policy makers and technical specialists identified the need for a functioning primary health care system, a feasible PNC visit schedule, community demand, a functioning system to notify CHWs of a birth, and a cadre of CHWs who are qualified, motivated, have adequate time, access and transport [7]. We identified no qualitative studies at community level. Such research could provide evidence on why visits may, or may not, occur based on the experiences of the providers and beneficiaries. This paper reports the findings of a study, conducted in Ethiopia, on factors affecting early postnatal home visits by CHWs - Health Extension Workers. This is particularly timely as a Community Based Newborn Care program is currently being rolled out across the country, which includes early postnatal contacts to identify and manage neonatal sepsis at community level, and to provide counselling to families on newborn care [15].

## Methods

### Program description

The Health Extension Program was introduced in 2003, and has provided one year of training to over 30,000 female Health Extension Workers (HEW). Two salaried workers, educated to at least grade 10, are selected by local councils to serve an area of around 5000 people. They are stationed in health posts and are supported by a network of community volunteers, called the Health Development Army (HDA). HEWs provide health promotion, and disease prevention and treatment, both in the community and at the Health Post [16–18]. In 2009 a program to equip HEWs with the skills to provide essential newborn care was introduced, which included early post natal visits [19], and Community Based Newborn Care (CBNC) was added in 2014 including the identification and treatment of sepsis at community level.

### Study setting selection and characteristics

The study was conducted in the Amhara and Southern Nations, Nationalities and Peoples (SNNP) regions of Ethiopia. The study was conducted in areas where the Health Extension Program, including CBNC activities, are supported by The Last Ten Kilometers programme (L10 K), a project implemented by JSI Research & Training Institute, Inc. In addition to routine HEW activities, L10 K has implemented a Community Based Data for Decision Making platform that includes tracking pregnant women, recording their service needs and linking them with the health system, and Family Conversations to promote key maternal and newborn health messages [20–23].

We were interested in understanding barriers and facilitators to early postnatal care within a reasonably functioning system, and L10 K provided us with a list of *woreda* (districts) and *kebeles* (lowest administrative units) that they considered had a functioning HEW and HDA system. From this list we selected two typical *woreda* in each region, and one *kebele* in each *woreda* that was reasonably accessible to the data collection team. We refer to the selected *kebele*s as *kebele* “A-D” to maintain anonymity. Table 1 shows the characteristics of the selected *kebeles*, all of which had a predominantly subsistence farming based economy. There were no reports of the use of the private sector by any study participants and we did not identify any private providers of PNC in the study areas.

**Table 1:** Characteristics of Study *kebele*

Region	*kebele*	Ethnicity	Predominant religion	Access to health centers	Terrain
Amhara	*kebele* A	Amhara	Orthodox Christian	Moderate	Hilly
	*kebele* B	Amhara	Orthodox Christian	High	Hilly
SNNPR	*kebele* C	Gamo/Wolaita	Protestant/Orthodox Christian	High	Flat, physical area of *kebele* was large
	*kebele* D	Silte	Muslim	Moderate	Flat

### Data collection

Data were collected from end of March, 2015 to May, 2015. Ethical approval was gained from the research ethics committees of the London School of Hygiene and Tropical Medicine, and from the Ministry of Science and Technology in Ethiopia. Written informed consent was obtained from all respondents. Data were collected using pre-tested semi structured guides developed by the authors. Data were collected as part of a wider study on the mechanisms of behaviour change related to key maternal and newborn care practices.

Data were collected from recent mothers, grandmothers, fathers, HEWs and HDA members using narrative and in-depth interviews, friendship pair interviews and focus group discussions. Collecting data from several respondent groups, and using a range of methods, ensured we captured a range of views and allowed for data triangulation. Sample sizes, respondent groups, the rationale for using each method, and the content related to early PNC visits are shown in Table 2. Sample sizes were based on prior experience of when saturation was likely to be reached. The possibility of conducting additional interviews and FGDs was built into the data collection plan in case saturation was not reached as anticipated.

**Table 2:** Data collection method rational and content

Method	Sample size	Rational	Content related to whether a PNC visit occurred
Narrative interviews with recent mothers	12	To collect in-depth data on personal experiences	• Whether the woman received a PNC visits and when • If a visit occurred, their perceptions of how the HEW knew they had delivered • If a visit did not occur their perceptions of why they were not visited
In-depth interviews with recent mothers	13	To collect in-depth data on perceptions and beliefs around what happens in their community	• The main roles of HEWs and HDAs in the community • Their perceptions of whether most people in the community receive a PNC visit, when and why • Whether they received a PNC visit and when
Friendship pair interviews with recent mothers and a close friend	5	To reduce social desirability bias by collecting data on experiences and beliefs in a comfortable and friendly setting in the presence of a friend	• Whether the recent mother received a PNC visit, when and why/why not
FGDs with mothers, grandmothers, fathers	4 with each group	To collect data on perceptions of what happens in their community through discussions that aim to enhance thinking about the topic	• Whether people in the community are visited, when, and why/why not
FGD with HEW and HDA	2 with each group	To collect data on what HEWs and HDAs do, problems and barriers that they face using methods that aim to enhance thinking about the topic and reduce social desirability bias	• Pile sort of activities done by HEW, including early PNC visit • Pile sort of activities they view as important and less important, including early PNC • Root tree analysis of why early PNC visits may not occur

The recent mothers and grandmothers were identified with the assistance of HEWs and HDAs and through snowball sampling. They were eligible for inclusion if they, or their daughter/daughter in law, received at least one home visit by an HEW or HDA in the ante or postnatal period. Recent mothers were purposively selected to ensure a range of ages, educational levels, parities, sex of newborn and socio-economic status that reflected the variation within the study site. For example, less than 14% of women in the study regions had secondary school education [24], and our purposive sampling focused on women with no or primary education. Place of delivery was also set as a selection criteria but it proved difficult to get many respondents who delivered at home. Fathers were recruited through the local authorities or through the HEWs and HDAs. All HDAs in a *kebele* were invited for the HDA FGDs. As there were only 2 HEWs per *kebele*, HEWs from neighbouring health posts were invited to the HEW FGDs. Selected respondents were approached in their home or place of work by the interviewers who explained the study, answered questions and took consent. Three participants refused: one wanted to attend a funeral, and the other two were too busy with their household chores to be able to participate.

Data were collected by four trained interviewers in the local language, with translators used in SNNPR. Interviews lasted from 1 to 2 h and took place in respondents’ houses. FGDs consisted of 3–7 participants and lasted from 1.5 to 2.5 h. They were conducted in neutral locations such as community centres. Interviews and FGDs were audio-recorded and fully transcribed in English within a week of data collection. During data collection, interviewers received regular feedback from senior researchers on their transcripts, interview techniques and to discuss emerging themes. The research team met twice during data collection to ‘pre-analyse’ the data in-order to document emerging themes and identify areas that needed to be explored in subsequent interviews and focus groups and to determine if saturation had been reached.

### Data analysis

We conducted a thematic analysis, this consisted of multiple readings of the postnatal care sections of the transcripts to ensure familiarity with the data, to get an understanding of the data as a whole, to begin to identify recurring ideas, and core, a-typical or notable ideas, − these were captured in reflective notes. General themes were derived deductively from the research aims, these were: Timing and content of PNC visits, reactions to PNC visits, and factors influencing the occurrence of visits. Analysis for the timing of, content and reaction to PNC was semantic/descriptive, while coding for factors influencing the occurrence of visits included examining the data for latent constructs. Interviews and focus groups were coded within the broad themes using NVIVO. Coding consisted of reading each transcript and identifying the underlying meaning of each segment of text. For each segment we considered what the segment was about, what message it was giving, what stuck out, and how it was different or similar to others segments, each segment was then given one or more code related to its underlying meaning. Codes that contained similar concepts were sorted and placed into themes. Themes and codes were refined and adjusted by looking for patterns, links and contradictions within themes. Data credibility was checked by triangulating data between respondent groups and between data collection methods. Data analysis was done by two of the senior researchers who discussed their coding regularly to enhance conceptual thinking and increased coding rigor.

## Results

There was considerable variability in the reported occurrence and timing of post-natal care (PNC) visits by HEW among the four study *kebele*. Respondents from all respondent groups consistently reported that HEWs in *kebele* A and B in Amhara visit most mothers within 3 days of giving birth. But the replacement of two longstanding HEWs by temporary workers in *kebele* B was reported to have led to a recent decline in visits. Visits were reported as occurring late or not at all in *kebele* C in SNNPR, and were said to be largely non-existent in *kebele* D in SNNPR. Reflecting their reportedly frequent occurrence, PNC visits in the Amhara *kebeles* were described as *‘ordinary’,* while in *kebele* D respondents had *‘never heard’* of this type of visit.

Through our inductive coding three main interlinked themes related to why early PNC visit may or may not occur emerged. These were physical issues related to accessibility and transport issues; information issues related to whether the HEW knew about the delivery; and work issues related to HEW availability, HEW performance and organization. We found no pattern in relation to PNC visits and maternal education level, with nearly all of the mothers interviewed having no or primary education levels.

### Physical issues

The main physical barriers to early PNC visits were related to accessibility (distance, spread of villages and topography) and to transport. In all study areas, there were households that were classed as inaccessible by all participants due to time or terrain issues. For example, in *kebele* A, there were some areas that were accessible only by ladders or only in the dry season, and these were difficult for the HEWs to visit:

*‘Some of the places are quite mountainous, and other places can only be accessed using a ladder to descend a ravine….There are places that we can’t access in the wet season…. Those that are nearer are not problematic. We get them within 24 hours’* [*kebele* A, Amhara, HEW – FGD].

Generally a flat topography and clustered settlements were seen to facilitate visits and a hilly terrain and scattered settlements were seen as a barrier. A flat terrain meant that households were generally accessible by transportation, which was facilitated by the few HEWs that had bicycles: *‘The topography of this kebele is flat like …. no ups and downs It is accessible for cars and walking’* [*kebele* D, SNNPR, Fathers – FGD].

Although extreme distances and terrains were limiting factors for PNC visits, moderate difficulties were not a limiting factor. *Kebele* A and B had the most difficult terrain overall, but these *kebele* are where community members reported that early PNC visits were most common. In contrast *kebele* D was generally flat and accessible but PNC visits were reported by community members as not occurring at all suggesting that information and work issues can be more important barriers than moderate physical difficulties.

### Information issues

Whether the HEWs’ knew a birth had occurred was a key theme in whether early PNC visits occurred. The main sub-themes were whether there was a functioning alert mechanism; and minor sub themes were place of delivery, migration in pregnancy and whether the pregnancy carried any stigma.

In the Amhara *kebeles* (A and B), where visits were reported as being common, mothers reported that HEWs knew they had delivered because they were involved in the delivery or in calling the ambulance. Women in these *kebeles* reported that they were told to inform the HEW when they went into labor, and few women had the ambulance number themselves: ‘*I directly went to their office [health post] when I felt labor pain, and it is the HEWs who called for Ambulance services’* [*kebele* A, Amhara, mother-narrative]. In the difficult to access areas of these *kebeles*, HEWs reported that they relied on the HDAs informing them of the delivery, which was hampered by accessibility issues as HDAs went in person or sent a messenger to inform an HEW of a delivery:

*‘The problem is that we do not get the feedback through the HDAs on time. They have to go a lot of distance and they sometimes send the feedback through students…….because of that we visit them after 7 days. So that is our major problem’* [*kebele* A Amhara, HEW-FGD].

In the SNNP *kebeles* (C and D), where PNC visits were reported as occurring late or not at all, women were given the ambulance number directly, often by the HDA. The HEWs thus relied on the HDA leaders informing them that a woman had given birth, and this did not always occur. In particular, HDAs in *kebele* D (where PNC visits were rare) reported that they did not always tell the HEWs about a delivery *‘The problem may be with us [HDA].….for example there was a mother that had delivered; I did go and visit her but I have not come and tell the HEW about it’* [*kebele* D, SNNPR, HDA – FGD]. This lack of provision of information was not linked to a lack of HDA activity, which was high in these *kebele*. Instead, there were some reports that the HDAs were tasked by the HEWs to conduct their visits and did not see any benefit of informing them of a delivery.

Other means of identifying delivered women were using the expected date of delivery, word of mouth and being informed by the family. Using the expected date of delivery was reported as problematic as the date were not accurate, and word of mouth was only useful where HEWs were very active in the community and made regular visits. In *kebele* A, in Amhara, and *kebele* C, in SNNPR, HEWs reported that the women themselves were meant to inform the HEW of the delivery, but mothers did not know that they should inform the HEW, and this mechanism was not functioning.

At the time of the study, few women in the study Kebles reported delivering at home, with major efforts from HEWs and HDAs to ensure all women delivered in a facility. Delivering at home was reported as shameful, with threats of sanctions and a fear that the HEW and HDA would be angry. Respondents from the mother, father and HEW/HDA FGDs all reported that HEWs would not know about women who delivered at home *‘If a woman gives birth at home, the HEWs won’t hear about it. Nobody tells them that she has given birth’* [*kebele* A, Amhara, Mother – FGD]. In the few cases we found where the HEWs knew about a home delivery, the women felt that they were denied PNC visits as a sanction for not delivering at a facility or not attending ANC, and this was reiterated in a mothers in the FGDs: ‘*If she [mother] refused and decided to stay at home, they [HEW] will never visit her and she just sit at her house alone….they won’t be at her side [kebele A, Amhara, Mother- FGD].* In general attending ANC was a proactive decision by the family, while the families’ roles in receiving PNC visits was passive, with no active care seeking decisions in relation to the home visits. We found no pattern or link between attending ANC and receiving a PNC visit.

Other minor themes related to HEWs being unaware of deliveries, that were reported in the HEW/HDA FGDs, were that some first time mothers travel to their own mother in another *kebele* to deliver, and that unmarried *‘teenagers’* keep their pregnancies and deliveries secret due to the stigma attached to them.

### Work issues

Two main sub themes related to work issues that affected whether timely PNC visits were made were: HEW availability and HEW work ethic and organization. Mothers, HEWs and HDAs reported workload and HEW availability as barriers to making timely PNC visits. Issues included being unavailable for visits as they were escorting women for delivery, multiple women delivering in different locations at the same time, staff absences, and participation in training workshops, meetings and health campaigns: *‘There are only two HEWs. They have lots of activities, which they are expected to perform. Therefore, they cannot cover all mothers in the three days after delivery’* [*kebele* C, SNNPR, Mother – FGD]. Some HEWs were more organized in terms of dividing up the community and having a clear plan for visiting communities, and these plans facilitated early visits.

Differences in HEW work ethic was also identified as a reason for no or late PNC visits, and the main differences between the *kebele* with reported high and low levels of early PNC visits was the general level of activity of the HEWs. In *kebeles* with low PNC visits HEWs were reported as only coming to the community for vaccinations, being rarely at the health post, or rarely leaving the health post: *‘They [HEWs] spend the whole day here [at health post] but no one come to here…..they didn’t go inside the village’* [*kebele* D, SNNPR, Father - FGD]. Temporary HEWs were reported as having particular issues with poor links with the community and with the HDA. This translated into few PNC visits being made: *‘Such staff [temporary] have a feeling that they will not be there in the kebele for long and show some sort of reluctance. They will not take their job … they believe that another person will take over from them very soon’* [*kebele* A, Amhara, HEW – FGD]. In *kebeles* with low PNC coverage the HEWs sometimes relied on strong HDA teams to conduct activities, or were reported as only being interested in ensuring facility deliveries occurred.

In contrast, in those *kebeles* with reported high PNC coverage, community members described a general high level of HEW activity and a sense of connectedness between the community and the HEW:

‘*They themselves live with us. They are with us when the baby is delivered. They visit us every day. They do not do only visiting within three days. They call for Ambulance; they escort us to the health facility and assist us in delivery’* [*kebele* B, Amhara, Mother - FGD].

## Discussion

Home visits by community health workers to provide postnatal care have the potential to reduce newborn mortality [5, 6], but observational data suggest that these need to occur within 24 h to be effective [8]. Despite the importance of PNC visits, achieving early visits in sub Saharan African settings has proved difficult [6, 9–11, 25], and there has been little research on why early visits are not achieved. We found that visits were affected by three inter-linked issues: physical issues (accessibility and transport), information issues (whether the HEW knows about the delivery), and work issues (HEW availability and performance).

Accessibility as a barrier to CHW performance has been found in several settings [26], and there were areas in all the study sites that were inaccessible due to topography or distance constraints. We found that moderate accessibility issues were not barriers to early visits if HEWs were active, organized and well connected to the community. Care should be taken when demarcating work areas to exclude areas that are unreachable; such areas may require their own CHW for timely visits to occur. The demarcation of small and realistic catchment areas was hypothesized as a reason why Village Health Workers (VHW) in a study in Bangladesh achieved a coverage of early home visits of 87% [13].

Knowing that a delivery has occurred was essential for HEWs making a timely PNC visit, and no or late notification of a delivery has been identified as a barrier to early home visits in other settings [8, 10, 13]. In the study areas formal notification strategies existed through HDAs informing HEWs of a delivery, although this did not always occur despite the existence of enhanced tracking of pregnant women through L10Ks’ Community Based Data for Decision Making platform. Timely notification by HDAs was hindered by distance, and HDAs did not report using mobile phones for notification. Providing mobile phones or air time could improve notification rates, with an association between having the HEWs phone number and receiving an early PNC visit found in a previous Ethiopian study [14]. Evidence suggests that using mobile phones to increase communication is highly valued by CHWs because it reduces travel time and enhances efficiency [27], however, this may be difficult where mobile phone coverage levels are low.

In two of the study *kebeles* families notified the HEWs directly when they went into labour, and HEWs played an active role in calling an ambulance or assisting with the delivery. This demonstrates that when families are aware of the need to notify a HEW, and have a reason to do so, then they are able and willing to do this. Our study suggests that family led notification, especially if done in labor, may result in earlier PNC visits than HDA led notification. Family led notification is likely to be enhanced by HEWs making pregnancy visits, especially if family members are included [10, 14], as this may improve links between the HEW and the family, and helps ensure that families know their role in notification. Facilities did not play a role in the notification process in the study *kebeles*. As rates of facility delivery increase facilities could play an important role in notification, particularly for newborns identified as high risk who may benefit most from early visits. The passive role that families currently play in PNC home visits may explain why we saw no pattern in visits by education levels, mirroring the lack of association between socio demographic characteristics and PNC visits found in other studies [10, 11, 14].

Issues with HEW availability were also reported as affecting PNC coverage, with competing tasks meaning that HEWs were not always available for timely visits. Such factors are largely out of control of the HEWs, and care needs to be taken to ensure that CHW tasks are feasible and take into account travel time. There was some evidence that HEWs and HDAs were focusing on facility deliveries to the detriment of their other activities.

The greatest difference between *kebeles* with high and low PNC coverage was the general activity level of the HEW. This affected notification issues and was more important than moderate physical barriers. In some *kebeles*, HEWs were reported as rarely coming to the community or relying on active HDAs to perform tasks, whilst in others they were reported as making frequent visits and were clearly well connected to the community. There was a particular problem with temporary workers. The role of motivation in performance is well recognized and numerous studies have reported on general motivators of CHWs. Few studies have explored variability in motivation across areas, nor the role of contextual issues, although these appear to be important [26]. We identified only one study that aims to rigorously evaluate methods of improving CHW motivation: the results of this study are yet to be reported and more research is needed [28].

We found little evidence of socio-cultural barriers from the community side for early visits, except that home delivery may be a barrier for notifying HEWs for fear for sanctions, and that some first time mothers travel to their home community for delivery. The use of sanctions for those who deliver at home may lead to vulnerable families not receiving vital services. There is much attention now being paid to the disrespect and abuse of women in facilities [29, 30]: there is also the potential for this to occur during home visits.

During this study we took several steps to maximize data quality and data transferability such as the use of multiple study sites, methods and respondent groups; the use of methods that aimed to overcome social desirability bias; purposive sampling to saturation; regular meetings and feedback during data collection to enhance reflective thinking; triangulation of data and team analysis. Despite this there is the potential for reporting and recall bias. Data were collected from small geographic areas that were accessible to the study teams and had reasonably functioning HEW systems, the findings may not apply to other areas with significantly different contextual issues, however the study findings suggest several issues that could be explored and considered when exploring issues related to PNC coverage in other settings. Studies in other settings in Ethiopia would further enhance transferability.

## Conclusion

If timely postnatal home visits are to occur, programs need to ensure that CHWs have realistic catchment areas that reflect their workload. Inaccessible areas may need their own CHW. Good notification systems are essential, families will notify CHWs if they have a clear reasons to do so, and more work is needed on how to ensure other potential notification systems function. Work ethic was a clear influencer on whether home visits occur, studies to date have tended to explore the motivation of CHWs as a group rather than exploring differences between CHWs, more studies on understanding motivation at an individual level are needed.
